# A Ratiometric Optical Dual Sensor for the Simultaneous Detection of Oxygen and Carbon Dioxide

**DOI:** 10.3390/s21124057

**Published:** 2021-06-12

**Authors:** Divyanshu Kumar, Cheng-Shane Chu

**Affiliations:** 1 Department of Mechanical Engineering, Ming Chi University of Technology, Taishan Dist., New Taipei City 243303, Taiwan; iamdk140@gmail.com; 2 Research Center for Intelligent Medical Device, Ming Chi University of Technology, Taishan Dist., New Taipei City 243303, Taiwan

**Keywords:** ratiometric, optical dual sensor, PtTFPP, CdSe/ZnS QDs, phenol red, PolyIBM

## Abstract

Simultaneous detection of carbon dioxide (CO_2_) and oxygen (O_2_) has attracted considerable interest since CO_2_ and O_2_ play key roles in various industrial and domestic applications. In this study, a new approach based on a fluorescence ratiometric referencing method was reported to develop an optical dual sensor where platinum (II) meso-tetrakis(pentafluorophenyl)porphyrin (PtTFPP) complex used as the O_2_-sensitive dye, CdSe/ZnS quantum dots (QDs) combined with phenol red used as the CO_2_-sensitive dye, and CdSe/ZnS QDs used as the reference dye for the simultaneous detection of O_2_ and CO_2_. All the dyes were immobilized in a gas-permeable matrix poly (isobutyl methacrylate) (PolyIBM) and subjected to excitation using a 380 nm LED. The as-obtained distinct fluorescence spectral intensities were alternately exposed to analyte gases to observe changes in the fluorescence intensity. In the presence of O_2_, the fluorescence intensity of the Pt (II) complex was considerably quenched, while in the presence of CO_2_, the fluorescence intensity of QDs was increased. The corresponding ratiometric sensitivities of the optical dual sensor for O_2_ and CO_2_ were approximately 13 and 144, respectively. In addition, the response and recovery for O_2_ and CO_2_ were calculated to be 10 s/35 s and 20 s/60 s, respectively. Thus, a ratiometric optical dual gas sensor for the simultaneous detection of O_2_ and CO_2_ was successfully developed. Effects of spurious fluctuations in the intensity of external and excitation sources were suppressed by the ratiometric sensing approach.

## 1. Introduction

The simultaneous occurrence of carbon dioxide and oxygen is quite common. Processes like photosynthesis, fermentation, respiration, etc. involve the evolution and consumption of both gases at the same time. In many such reactions related to biological research, medical analysis, and biogeochemical processes the simultaneous measurement of both of the gases are desirable and required to quantitatively control their concentrations [1,2,3].

In recent years, optical methods based on absorptiometry, reflectometry, infrared, fluorescence, and surface plasmon resonance have been frequently used for the development of single-analyte gas sensors [4]. Among these methods, the fluorescence-based optical method is typically used because of its high sensitivity and selectivity [5]. Several individual O_2_ and CO_2_ optical sensors based on this method have been reported [6]. Moreover, this method demonstrates the capability to develop multi-analyte sensors [7,8]. Here, the sensors are designed in such a manner that the fluorophores sensitive to the respective gases exhibit fluorescence signals at different wavelengths and are only responsive towards that particular gas. In addition, a suitable gas-permeable matrix is used to host the fluorophores that facilitate the penetration of gases. Several studies have already been published describing the development of dual O_2_–CO_2_ sensors based on the fluorescence lifetime or fluorescence intensity measurement methods [9,10,11,12,13]. Most of the previously developed O_2_–CO_2_ dual sensors use either a single- or double-layer approach to design the sensor system. In a single-layer designed sensor, both the O_2_- and CO_2_-sensitive fluorophores are incorporated in a common matrix and coated as one layer, whereas in double-layer sensors, the fluorophores are incorporated in different matrices and coated in separate layers one above the other. Nevertheless, the single layer design is simpler and more straightforward with rapid response due to a shorter diffusion distance [14]. Although multiple O_2_–CO_2_-based dual sensors with good sensitivity and response time have recently been developed, the practical use of such devices requires more precise and better sensor characteristics. Most of the currently available dual sensors exhibit mutual interference and poor selectivity [14]. The response–recovery times and sensitivities are good, but considerably better times and sensitivities are required. In addition, an intensity-based sensor is limited by several interferences due to temperature change, non-uniform distribution of indicator concentrations, intensity fluctuations, equipment-based errors, etc. Therefore, an effective dual O_2_–CO_2_ sensor with an appropriate simpler design and better performance is still not available.

Most of these errors can be eliminated using an appropriate referencing method, which have not been proposed thus far. One such method is fluorescence-based ratiometric referencing method, which is used herein [15,16]. In this method, an additional reference signal exhibited by another fluorophore is available in the designed sensor. The sensitivities of such a sensor are calculated as the ratio of the fluorescence intensity of the indicator dye to that of the reference dye at the respective analyte concentrations. Oxygen is paramagnetic and is a well-known collision quencher. In principle, O_2_ detection is based on the fluorescence quenching of the sensitive dyes by oxygen. A number of oxygen-sensitive dyes for oxygen detection are available [17]. Compared with the other dyes, metalloporphyrins, such as Pt and Pd complexes, are highly sensitive and selective for O_2_ [18,19,20,21]. Specifically, the dye platinum (II) meso-tetrakis(pentafluorophenyl) porphyrin (PtTFPP) exhibits outstanding optical properties, including a large Stokes shift, excellent photostability, narrow emission bandwidth, and a higher quantum yield, making it highly suitable for the development of a more efficient oxygen sensor. Such indicators are doped into a suitable gas-permeable matrix, such as a polymer or sol–gel matrix, for enhancing molecular contact to achieve higher sensitivities. Therefore, in this study, PtTFPP doped into a polymer matrix poly(isobutyl methacrylate) (PolyIBM) was used as an O_2_-sensitive dye.

On the other hand, gaseous CO_2_ is measured by infrared absorptiometry, as well as electrochemically using the Severinghaus electrode, and pH indicators [6,22]. CO_2_ measured by using pH indicators exhibits several advantages over the others including higher sensitivity and better linearity with quick response and recovery times. pH indicators are weak acids or bases that undergo a reversible change in their optical properties (absorbance or fluorescence) due to their deprotonation and protonation. Recently developed optical CO_2_ sensors exploit a colorimetric change in pH indicator dyes, such as thymol blue, phenol red, and α-naphtholphthalein, or a fluorescence change in luminescent dyes such as 1-hydroxypyrene trisulfonate (HPTS) and fluorescein derivatives to detect CO_2_ [23,24,25,26]. However, colorimetric-based pH indicators exhibit less sensitivity, and fluorescent pH indicators are very limited. Very recently, few studies have reported where the combination of a colorimetric pH indicator and a luminescent dye as an internal reference dye is utilized to develop CO_2_ sensors with higher sensitivities [27]. Here, the luminescence band of the internal reference dye overlaps with the absorption band of the pH indicator, which is altered by the absence and presence of CO_2_. Thus, in the presence of CO_2_, because of resonance energy transfer (FRET), the fluorescence intensity of the internal reference dye changes, indicating the presence of CO_2_. One such sensor has been used to develop a CO_2_ sensor, which combines the use of α-naphtholphthalein and tetraphenyl porphyrin (TPP) as the pH indicator and internal reference dye, respectively [28]. Furthermore, polymer matrixes such as PVC, PVA and sol–gels have been used to provide an appropriate microenvironment for the fluorescent molecules. It stabilizes the acid or base form enhancing the gas diffusivity and surface contact between the molecules, leading to a more rapid response and recovery towards CO_2_. In this study, the combination of Phenol red (pH indicator) and CdSe/ZnS (A570) QDs (internal reference dye) was used as a CO_2_-sensitive system. The excellent photophysical properties of CdSe/ZnS (A570) QDs and the large extent overlap of its emission band with the absorption band of phenol red made it suitable to use as an internal reference dye.

Table 1 shows the material used for optical O_2_/CO_2_ dual sensors and compares the performances of current sensors with that of different sensor types fabricated using various O_2_/CO_2_-sensitive dyes. On the other hand, a number of researches have presented the fluorescence intensity and lifetime based on single/dual layer methods. In this study, we describe an optical dual sensor based on the fluorescence ratiometric referencing technique using a single-layer approach. This work utilizes the PtTFPP complex for O_2_ sensing, the combination of CdSe/ZnS (A570) QDs and phenol red with tetraoctylammonium hydroxide (phase-transfer reagent) for CO_2_ sensing, and CdSe/ZnS (A460) QDs for ratiometric measurements. All of the fluorophores were incorporated in a polymer matrix Poly(IBM) and coated on a plastic membrane. The developed dual sensor in this study can be utilized in environmental air quality monitoring, gas measurements in biological processes in tissues.

## 2. Experiments

### 2.1. Materials

PtTFPP was purchased from Frontier Scientific (Logan, UT, USA). CdSe/ZnS (A560), and CdSe/ZnS (A460) were purchased from Taiwan Nanocrystals Inc. (Tainan City, Taiwan). Phenol red and Poly(IBM) were obtained from Alfa Aesar and Scientific Polymer Inc., respectively. Tetraoctylammonium hydroxide (TOAOH) was purchased from Aldrich and synthesized following a reference. In addition, toluene was purchased from Tedia Company Inc.

**Figure 1:**
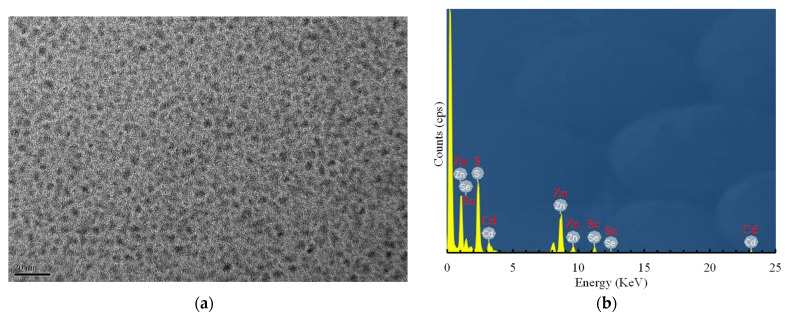
(**a**) TEM image of CdSe/ZnS QDs at a resolution of 20 nm and (**b**) EDX analysis results for CdSe/ZnS QDs.

Figure 1a,b shows the TEM image of the CdSe/ZnS QDs and their corresponding energy-dispersive X-ray spectroscopy (EDX) analysis results, respectively. The CdSe/ZnS QDs mainly comprise Cd, Se, Zn, and S, where the x and y axes represent the energy (keV) and counts per second per electron (basically X-ray intensity), respectively.

### 2.2. Preparation of O_2_- and CO_2_-Sensing Materials

Initially, 200 mg of Poly (IBM) was mixed in 2 mL of toluene and stirred for 10 min at room temperature. After it dissolved completely, 30 mg of the CdSe/ZnS (A570) QDs was added to the solution and subjected to ultrasonication for 10 min at room temperature. An additional 20 mg of CdSe/ZnS (A460) was added, followed by ultrasonication for 10 min to obtain a clear solution. Subsequently, 0.5 mg of the PtTFPP complex and 1.5 mg of Phenol red is mixed in the solution and stirred. Finally, 100 µL of TOAOH is added and thoroughly stirred to obtain the final mixture. Sixty microliters of the final mixture were drop-coated on a plastic transparent membrane and dried in an ambient atmosphere for 15–20 min. The plastic membrane used here is composed of Polypropylene which is more resistive to the chemicals and organic solvents. The as-obtained sample as placed diagonally in a rectangular sample holder, which was connected to an LED light source from one side and a spectrometer detector on the adjacent side.

### 2.3. Instrumentation

**Figure 2:**
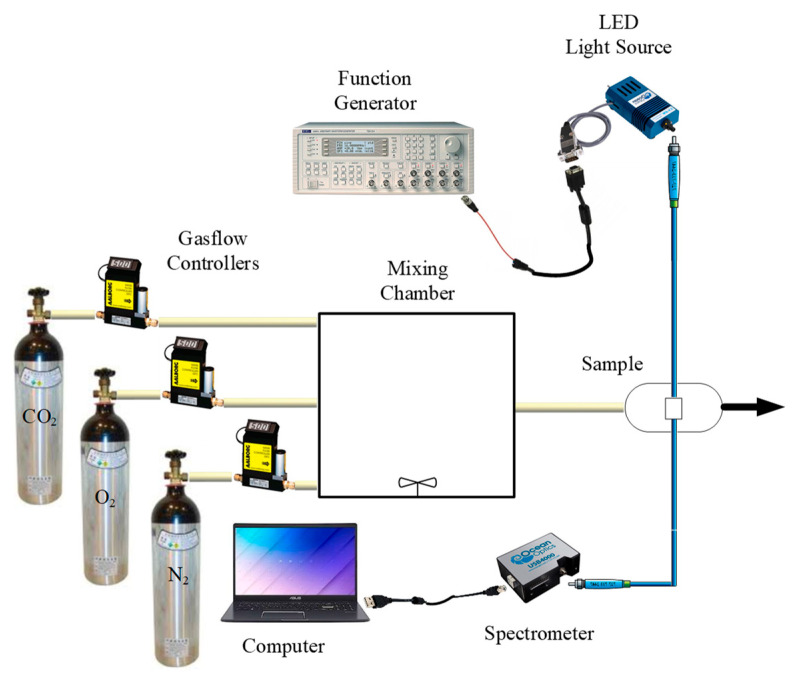
Schematic diagram showing experimental arrangement used for characterization.

Figure 2 shows the experimental set-up used for the performance characterization of the ratiometric optical dual sensor. A 380-nm central-wavelength LED light source driven by an arbitrary wave function generator (TGA1240, Thurlby Thandar Instruments (TTi) Ltd., Huntington, UK) at a frequency of 10 kHz was utilized for fluorophore excitation. The relative fluorescence intensities were measured using a USB 4000 spectrometer (U.S. Ocean Optics Inc., Largo, FL, USA). The gas flow was controlled using mass-flow controllers (Aalborg Instruments and Controls Inc., Orangeburg, NY, USA, Model GFC 17) and was mixed in a gas-mixing chamber. Absorption spectra of the fluorophores were recorded on a UV-Vis spectrophotometer.

## 3. Results and Discussions

### 3.1. Optical Properties of Dual Sensor

**Figure 3:**
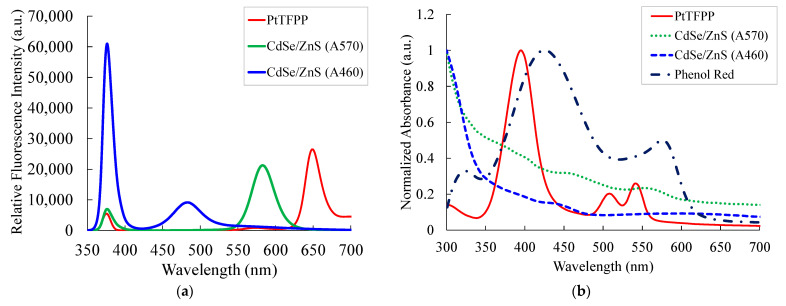
(**a**) Emission and (**b**) absorption spectra of PtTFPP, CdSe/ZnS (A570), and (A460) QDs.

Figure 3a,b respectively shows emission and absorption spectra of the different fluorophores (viz. PtTFPP, CdSe/ZnS (A570), (A460), and phenol red). Absorption spectra and emission spectra of each material were individually recorded using a thin film. The obtained absorption spectrum reveals that all of the fluorophores can be excited easily by an LED with a central wavelength of 380 nm. Clear and distinct emissions attributed to the O_2_ indicator PtTFPP, CO_2_ indicator CdSe/ZnS QDs (A570), and reference fluorescent material CdSe/ZnS QDs (A460) are observed, with corresponding central peak wavelengths at 650, 570, and 460 nm, respectively, using an LED light source with 380 nm for excitation (Figure 3b). In addition, the figure reveals that the absorption spectrum of Phenol red considerably overlaps the emission spectrum of CdSe/ZnS (A570) QDs, a condition ideal for FRET. Therefore, this combination can be effectively used for CO_2_ detection. Thus, the individual detection of gases is performed via the monitoring of well-resolved emissions of sensing materials.

### 3.2. O_2_ Sensing Properties of the Optical Dual Sensor

**Figure 4:**
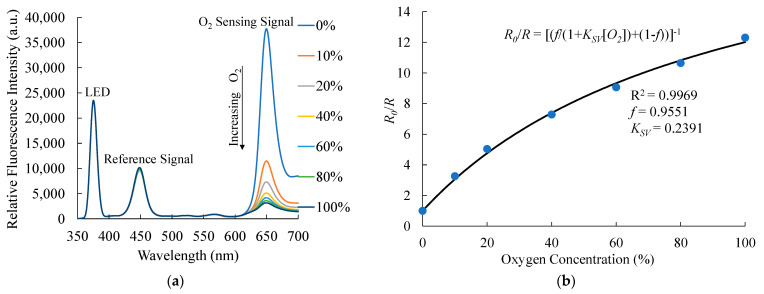
(**a**) Response of the optical dual sensor under exposure to different O_2_ concentrations and (**b**) Stern-Volmer plots.

Figure 4 shows the fluorescence intensity response of the dual sensor in the presence of different O_2_ concentrations. As expected, the fluorescence intensity of PtTFPP at 650 nm is selectively quenched considerably. With the increase in applied O_2_ concentration up to 100%, the quenching of the fluorescence intensity increases continuously, confirming that intensity quenching is proportional to the applied concentration. At higher O_2_ concentrations, the presence of increased number of oxygen molecules causes a larger number of molecular collisions and hence an increased extent of quenching. Moreover, the maximum intensity quenching is observed at low O_2_ concentrations, indicating that the sensor exhibits higher sensitivity at low O_2_ concentrations. Notably, intensities of the other fluorescence signals are unaffected or exhibit a negligible effect in the presence of O_2_, facilitating the detection of CO_2_ without any interferences. The response of the ratiometric sensor can be evaluated by *R*_0_ and *R* in the Stern–Volmer equation [33]:

(1) $$R_{0}/R={\left[f/(1+K_{SV}[O_{2}])+(1-f)\right]}^{-1}$$

where *R*_0_ and *R* represent the luminescence signal ratio of the sensor in the absence and presence of oxygen, respectively. *F* represents the fractional contribution to the total emissions, and *K_SV_* is the Stern–Volmer quenching constant. Figure 4b shows the plot between the ratiometric sensitivity (*R*_0_/*R*) and O_2_ concentration. With the increase in the O_2_ concentration, the ratiometric sensitivity of O_2_ increases and attains the maximum value of 13 at 100% O_2_. The downward curvature of the sensitivity plot can be explained by the modified Stern–Volmer equation shown in Equation (1). Moreover, the sensitivity plot is steeper at lower O_2_ concentrations (up to ~20%), indicative of the highly sensitive nature of the sensor at lower concentrations.

### 3.3. CO_2_ Sensing Properties of the Optical Dual Sensor

**Figure 5:**
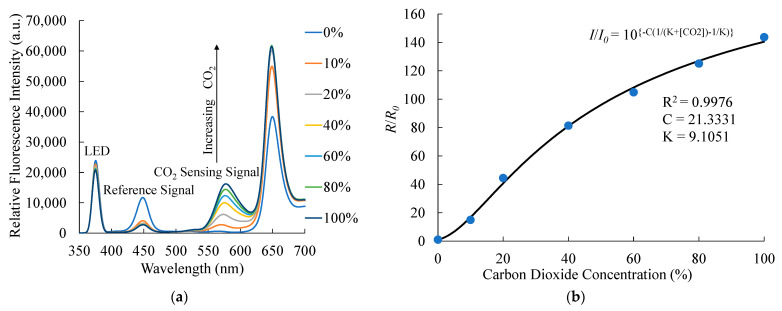
(**a**) Response of the optical dual sensor under exposure to seven CO_2_ concentrations and (**b**) Variation of *R*/*R*_0_ with 0-100% CO_2_ concentrations.

Similarly, Figure 5 shows the changes in the fluorescence signals upon exposure of the optical dual sensor to different CO_2_ concentrations. As expected, with the increase in the CO_2_ concentration from 0% to 100%, the fluorescence intensity of CdSe/ZnS (A570) QDs at 570 nm considerably increases. The intensity change is proportional to the applied concentration according to the Stern–Volmer equation. In principle, with the increase in the CO_2_ concentration, the absorption of phenol red at 570 nm decreases; hence, the observed fluorescence intensity of QDs increases. The relationship between the observed emission intensity of the CO_2_-sensing material and CO_2_ concentration follows Equation (2) [34]:

(2) $$R/R_{0}=10^{\left\{-C(1/(K+[CO_{2}])-1/K)\right\}}$$

where C is a constant and K is the equilibrium constant. With the increase in the concentration, the CO_2_ sensitivity increases, exhibiting the maximum value at 100% of CO_2_. The calibration plot in Figure 5b reveals the relationship between ratiometric sensitivity (*R*_0_/*R*) and the different CO_2_ concentrations by Equation (2). The ratiometric sensitivity of the optical dual sensor for CO_2_ is estimated to be 144.

### 3.4. Response Time of the Optical Dual Sensor

**Figure 6:**
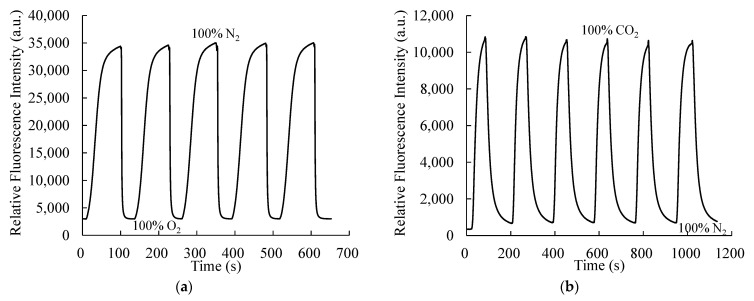
Response and recovery plots of the optical dual sensor for (**a**) O_2_ and (**b**) CO_2_.

For practical applications, sensors should exhibit a rapid response and recovery. Herein, the response and recovery of the fluorescence signals for the respective gases are individually calculated. The response and recovery represent the time taken by the optical sensor to achieve 90% of its final intensity and 90% of its initial intensity, respectively. For O_2_ response and recovery, the optical dual sensor is initially placed under 100% N_2_ and then switched to 100% O_2_ for 30 s, followed by switching again to 100% N_2_ for 60 s; this process is repeated for five cycles. Figure 6a shows the obtained plot. Figure 6a shows the response and recovery characteristics of the optical dual sensor for around 650 s in the presence and absence of O_2_. The PtTFPP complex completely recovers its fluorescence intensity after complete quenching at 100% O_2_. The response time taken for O_2_ to quench 90% of the intensity of PtTFPP complex is 10 s, while that taken to recover 90% of the quenched intensity is 35 s. Similarly, the response and recovery of the optical dual sensor for CO_2_ is calculated. Herein, the optical dual sensor is switched to 100% CO_2_ from 100% N_2_ and maintained for 60 s and then again switched back to 100% N_2_ for 90 s. Figure 6b shows the results where this process is repeated for six complete cycles over 1200 s. The corresponding response and recovery times for CO_2_ are calculated to be 20 and 60 s, respectively.

### 3.5. Dynamic Response of the Optical Dual Sensor

**Figure 7:**
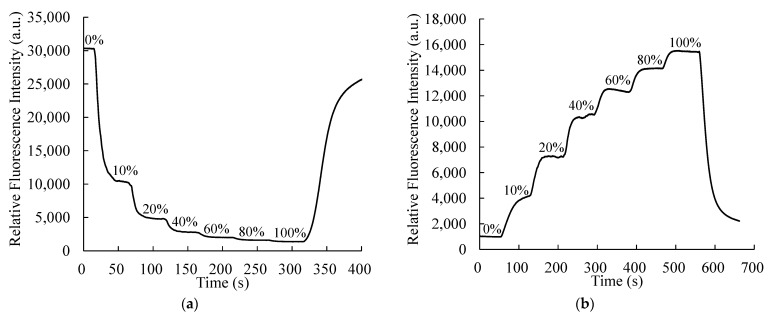
Dynamic response times of the optical dual sensor for (**a**) O_2_ and (**b**) CO_2_.

Dynamic response and recovery data for the optical dual sensor are also recorded with a pattern similar to that used in the above section. Figure 7a,b shows the dynamic response and recovery for O_2_ and CO_2_, respectively. By switching the dual sensor from 100% N_2_ to 10% O_2_, the response and recovery times are calculated to be 15 and 27 s, respectively. The corresponding response and recovery times for CO_2_ are 34 and 42 s. Thus, the optical sensor also exhibits a rapid response and recovery at low concentrations, which makes it suitable for low-level detection.

### 3.6. Selectivity of Optical Dual Sensor

**Figure 8:**
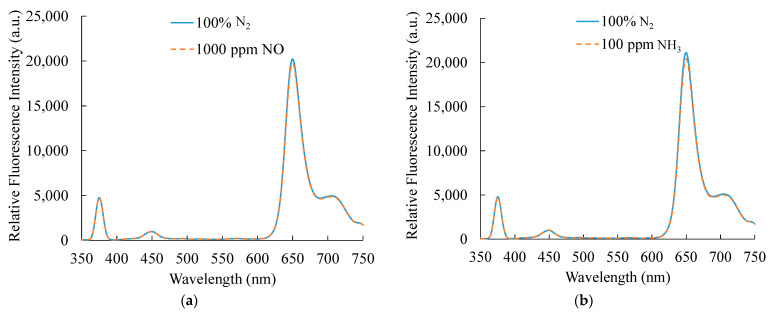
Responses of the optical dual sensor towards (a) NO and (b) NH_3_.

The presence of certain other gases under practical conditions sometimes creates interference in the appropriate operation of the gas sensor. Therefore, the selectivity of a gas sensor is also a key property to be considered. Herein, the response of the ratiometric dual sensor in the presence of nitric oxide (NO) and ammonia (NH_3_) was investigated. The proposed dual sensor is alternately exposed to both gases for 15 min to observe changes in fluorescence intensities. Figure 8 shows the obtained results. Under 1000 ppm NO, the fluorescence intensities of the sensor exhibit an almost negligible change. Similarly, changes are not observed in the presence of 100 ppm NH_3_. These results confirm that the proposed ratiometric dual sensor does not exhibit any interference by NH_3_ and NO.

### 3.7. Cross Sensitivity of the Optical Dual Sensor

**Figure 9:**
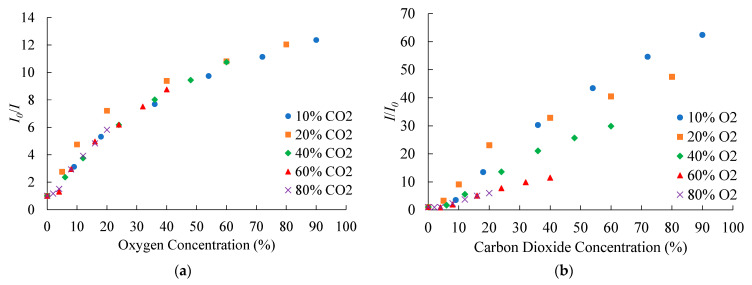
Cross-sensitivity calibration of the dual sensor for (**a**) O_2_ and (**b**) CO_2_.

The ratiometric dual-sensor does not show cross-sensitivity while a substantial change in the intensity of O_2_ sensitive material at 650 nm is observed (Section 3.3) in the presence of CO_2_. This observed cross-sensitivity arises due to a change in the absorption part of phenol red which is overlapped with the emission of the PtTFPP complex. Therefore, a cross-sensitivity calibration for the ratiometric dual sensor is required for its practical applications. We have calibrated the response of the PtTFPP complex at different fixed CO_2_ concentrations. At each fixed CO_2_ concentrations, the fluorescence response of the PtTFPP complex at different O_2_ concentrations is recorded. At each fixed O_2_ concentrations, the fluorescence response of the CdSe/ZnS QDs (A570) at different CO_2_ concentrations is also recorded. The corresponding ratiometric sensitivities for O_2_ and CO_2_ are also calculated and plotted as shown in Figure 9a,b, respectively. The figure displays the ratiometric sensitivities for O_2_/CO_2_ at 10%, 20%, 40%, 60%, and 80% of fixed CO_2_/O_2_ concentrations. The sensitivity calibration plot shows a decrease in CO_2_ sensitivity with increasing fixed O_2_ concentration with a minimum obtained at 80% of fixed O_2_ concentration.

### 3.8. Humidity and Temperature Effect of the Optical Dual Sensor

**Figure 10:**
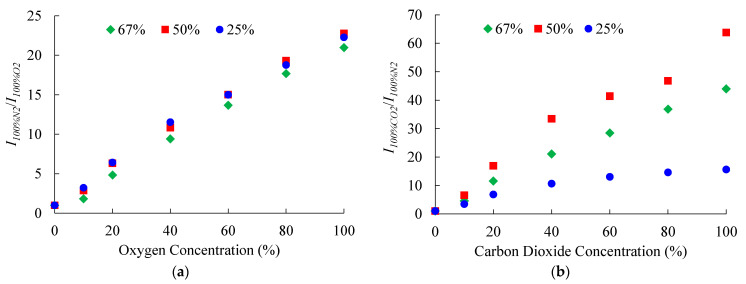
Effect of humidity on the sensitivities of (**a**) O_2_ and (**b**) CO_2_ of the optical dual sensor.

The ratiometric dual sensor is placed in different humid environments to observe changes in sensitivities for O_2_ and CO_2_. Figure 10 plots the sensitivities calculated at three values of relative humidity (RH) for different gaseous concentrations. The results in Figure 10a reveal no significant changes in the O_2_ sensitivities, while the CO_2_ sensitivity increases with the decrease in the RH value from 67% to 50%. However, with the further decrease in RH to 25%, the sensitivity decreases to its lowest value.

**Figure 11:**
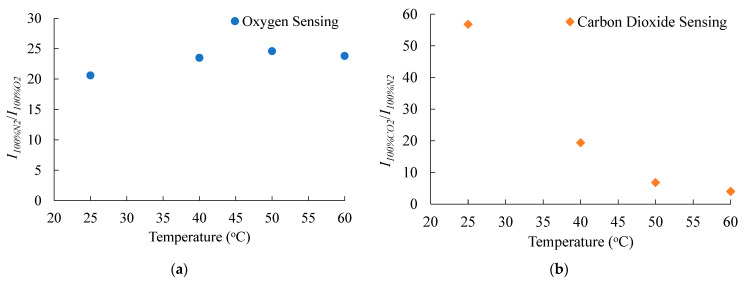
Effect of temperature on the sensitivities of (**a**) O_2_ and (**b**) CO_2_ of the optical dual sensor.

Previous studies have reported that gas sensors are extremely sensitive to temperature changes. In this section, the effect of temperature changes on the sensitivities of the dual sensor is examined. Figure 11 shows the relationship between sensitivities of O_2_ and CO_2_ at different elevated temperatures. With the increase in the working temperature from 22 to 60 °C, the sensitivity of O_2_ does not exhibit a significant change, whereas the CO_2_ sensitivity decreases from 58 to 5.

## 4. Conclusions

This work presents a new optical dual sensor for the simultaneous detection of O_2_ and CO_2_ based on the fluorescence ratiometric referencing method. The dual sensor employed PtTFPP as the O_2_ indicator, a combination of CdSe/ZnS (A570) and phenol red as the CO_2_ indicator, and CdSe/ZnS (A460) as the reference signal. The combine sensing materials can be excited by the same LED with a central wavelength of 380 nm, and their bright fluorescence has no spectral overlaps or cross-talk. The experimental result revealed the ratiometric sensitivity for O_2_ is 13 with a response and recovery times of 10 and 35 s, respectively, while the corresponding sensitivity for CO_2_ is found to be 144 with a response and recovery times of the 20 and 60 s, respectively. The proposed dual sensor makes it possible measure O_2_ and CO_2_ simultaneously more precisely. The ratiometric optical dual sensor developed in this study is insensitive to fluctuations in excitation and external light intensities.

## Figures and Tables

**Table 1:** Properties of typical optical O_2_ and CO_2_ dual sensors.

O_2_ Probe	CO_2_ Probe	λ_ex_ (nm)	λ_em_ (nm) O_2_/CO_2_	O_2_/CO_2_ Range	Response	Sensing Type	Reference
RTDP	HPTS	460	630/520	0–20 kPa/0–20 kPa	None	Dual layer/Intensity	[8]
PtTFPP	HPTS and Ir_2_(C30)_4_Cl_2_	525 (O_2_) 470 (CO_2_)	630/580	0–20%/0–18%	O_2_:30 s CO_2_:3 min	Dual layer/Lifetime	[9]
Ru(II)	(TOA)_3_HPTS	460	605/512	0–210.6 hPa/0–25.1 hPa	None	Single layer/Lifetime	[10]
PtTFPP/AFC	HPTS	405	650/487	0–100%/0–100%	O_2_:15 s CO_2_:7 s	Dual layer	[11]
PtOEP	HPTS	470	645/515	0–10 kPa/0–61 kPa	O_2_:5 s CO_2_:None	Intensity	[13]
Ir(II)	HPTS	450	560/520	0–20 kPa/0–4 kPa	O_2_:19 s CO_2_:49 s	Dual layer /Intensity and Lifetime	[29]
Ru(II)	HPTS	470	620/520	0–20%/0–8%	None	Single layer/Lifetime	[30]
PtOEP	Ru(II) and m-cresol purple	470	646/630	0–210 hPa/0–200 hPa	None	Not available/Lifetime	[31]
PtOEP	PtOEP and α-naphtholphthalein	525	646/646	0–30%/0–100%	O_2_:31 s CO_2_:31 s	Not available/Lifetime	[32]
PtTFPP	CdSe/ZnS and Phenol red	380	650/575	0–100%/0–100%	O_2_:10 s CO_2_:20 s	Single layer/Intensity (ratiometric)	This work

## Data Availability

Not applicable.
